# „… move from this hellish life!“: Interviewstudie zur Situation junger Menschen in afghanischen IDP-Camps

**DOI:** 10.1007/s12054-022-00484-1

**Published:** 2022-05-06

**Authors:** Susanne Leitner, Saleem Jalalzai

**Affiliations:** Reutlingen, Deutschland

**Keywords:** Afghanistan, Interviewstudie, Flüchtlingslager, Binnenvertreibung

## Abstract

Dieser Text gibt Einblicke in die Lebenssituation (junger) Menschen, die als Binnenvertriebene in Camps in und um Kabul leben. Anhand von Interviews mit Helferinnen vor Ort werden insbesondere existentielle Notlagen sowie besondere Gefährdungen für Mädchen und Frauen dargestellt.

Die Machtübernahme durch die Taliban veränderte die Lebensbedingungen vieler Menschen in Afghanistan erheblich. Ein Großteil der Bevölkerung ist von massiver Armut betroffen. Besonders Menschen, die im Zuge der Gewalthandlungen ihr Zuhause verlassen mussten und als Binnenvertriebene in provisorischen Camps gelandet sind, leiden existentielle Not. Auch angesichts anderer Krisen und Konfliktherde weltweit, sind wir im Sinne internationaler Solidarität gehalten, diese Menschen in Afghanistan nicht aus dem Auge zu verlieren.

Am 15. August 2021 eroberte die radikalislamistische Terrorgruppe der Taliban die afghanische Hauptstadt Kabul und versteht sich nach der Flucht des bis dato amtierenden Präsidenten Ashraf Ghani als Regierung (vgl. Al Jazeera 2021a^1^). Der endgültigen Machtübernahme in Kabul waren Eroberungszüge in mehreren Provinzen vorausgegangen, die Tausende von Menschen dazu bewegt hatten, ihre Heimatorte zu verlassen und unter anderem Schutz in der lange als sicher geglaubten Hauptstadt zu suchen. Viele von ihnen leben als Binnenvertriebene (*Internally Displaced Persons*, IDPs) in provisorischen Camps (vgl. OCHA Services 2021a). Die große mediale und gesellschaftliche Aufmerksamkeit, die die Situation in Afghanistan in den ersten Wochen unmittelbar nach dem genannten Ereignis auch in Deutschland erfuhr, scheint mittlerweile wieder abgeebbt zu sein. Gleichwohl ist es, so meinen wir, gerade für die Soziale Arbeit als menschenrechtsbasierte Profession (vgl. Prasad 2018) von Bedeutung, an dieser Stelle sensibel und informiert zu bleiben. Judith Butler (2021) betont, dass Menschen als grundsätzlich vulnerable Wesen von sozialer Interdependenz geprägt sind, woraus sie eine globale ethische Verantwortung füreinander ableitet. Dieses kann, so meinen wir, nicht nur für das allgemein Menschliche gelesen werden, sondern auch als eine Aufforderung zur Solidarität mit den hier interviewten Helfer_innen, die Soziale Arbeit leisten, wo institutionell keine Soziale Arbeit vorgesehen ist: zum Beispiel bei der Versorgung von Binnenvertriebenen in Kabuler IDP-Camps.

## Binnenvertriebene in IDP-Camps

Als Binnenvertriebene verstehen wir Menschen, die innerhalb ihres Herkunftslandes, also ohne Grenzüberschreitung zwangsmigrieren (vgl. Koch 2020). Koch (2020) konstatiert, dass Binnenvertriebene

häufig ähnlich schutzbedürftig wie grenzüberschreitende Flüchtlinge [sind, SJ&SL], haben aber keinen Anspruch auf internationalen Schutz. Ungeachtet der alarmierenden Zahlen mangelt es an politischer Aufmerksamkeit für Probleme, die aus Binnenvertreibung entstehen. (S. 5)

Die sogenannten IDP-Camps haben sich in Parks und an öffentlichen Plätzen in und um Kabul formiert, so zum Beispiel in Shahr-eNaw, einem zentralen Stadtteil mit vielen Supermärkten und (damals noch) internationaler Infrastruktur. Menschen aus ganz Afghanistan, die im Zuge von Kriegshandlungen, die der Machtübernahme durch die Taliban vorausgegangen waren, obdachlos geworden sind, hatten sich auf den Weg in die Hauptstadt gemacht, da diese Gerüchten zufolge Sicherheit bieten sollte. Sie ließen sich mit dem, was sie am Leibe hatten, nieder und bauten provisorische Zelte aus Tüchern. Da der Zulauf vieler Menschen in die so entstehenden und größer werdenden informellen Camps bei der Kabuler Bevölkerung nicht unbemerkt blieb, begannen wohlhabende Privatmenschen und lokale Hilfsorganisationen trotz der chaotischen Situation, eigenintiativ Spenden zu sammeln und die Menschen notdürftig zu versorgen. Bis heute fehlt es aber an einer zentralen Organisation und Verwaltung der Camps. Hilfsorganisationen auf nationaler Ebene, die von der alten Regierung gefördert wurden, haben zudem keinerlei Geldmittel zur Verfügung und sind auf das Ausland sowie auf private Spenden angewiesen. Aus diesem Grunde sind auch Gewaltschutzmaßnahmen, z. B. insbesondere der Schutz von Frauen (vgl. Krause und Schmidt 2018), nicht vorhanden^2^.

**Figure 1:**
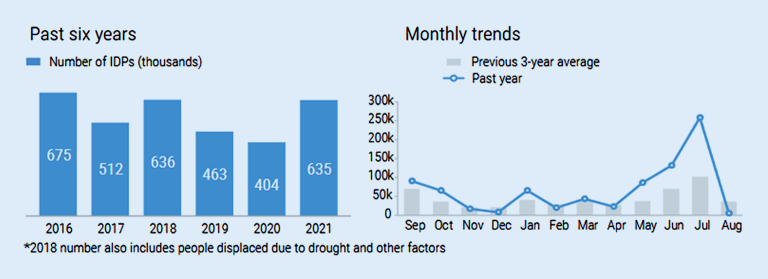

Humanitarian Response (2021) geht von rund 635.000 Menschen aus, die im Jahr 2021 als Binnenvertriebene in Afghanistan leben und beobachten eine massive Zunahme in den Sommermonaten selbigen Jahres. 59 % dieser Menschen sind nach diesen Zahlen Kinder unter 18 Jahren. Einschränkend muss gesagt werden, dass die Datenerhebung aufgrund der chaotischen Situation schwierig ist und die Zahlen zwischen Juli und September 2021 zum Teil durch Extrapolationen zustande gekommen sind (vgl. Humanitarian Response 2021; vgl. auch Abb. 1).

Zur aktuellen humanitären Situation zitiert der Nachrichtensender Al Jazeera (2021b) einen Bericht des UN Office for the Coordination of Humanitarian Affairs (OCHA): „Nearly all lack adequate shelter, access to medical care and sufficient food“ (Al Jazeera 2021b).

## Armut und Hunger

**Figure 2:**
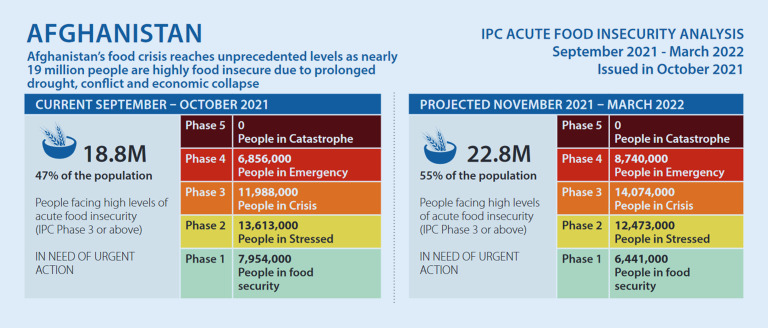

Diese Situation trifft auf eine allgemein hohe Armutsrate im Land. So leben laut Medico International zwischen 70 und 90 % der Menschen in Afghanistan unterhalb der Armutsgrenze, rund die Hälfte sei von Hunger bedroht (vgl. Rudhof-Seibert 2021). Auch die Organisation Integrated Food Security Phase Classification (IPC) (2021) geht davon aus, dass derzeit, mit steigender Tendenz, rund die Hälfte der afghanischen Bevölkerung ernsthaft Hunger leidet („high levels of acute food insecurity“, IPC 2021). Die vielseitigen und komplexen Gründe sind wohl vor allem in „forty years of war, recurrent natural disasters, chronic poverty, drought and the COVID-19 pandemic“ (OCHA Services 2021b, vgl. auch Abb. 2) zu sehen.

Aufgrund persönlicher Verbindungen bekamen wir (SJ & SL) zunächst informelle Augenzeugenberichte über die Lage in den IDP-Camps. Durch Kontakt zu in Kabul ansässigen Menschenrechtsaktivist_innen, die vor Ort Hilfe in IDP-Camps leisten, war es möglich, einen qualitativen Forschungszugang zur Situation der sich dort befindlichen Menschen zu generieren, wobei ein besonderes Augenmerk auch auf Kinder und Jugendliche gelegt wird.

## Sampling und Datenerhebung

Dieses Projekt entstand in Co-Autor_innenschaft, bei der eine Person Erfahrung mit erziehungswissenschaftlich orientierter qualitativer Sozialforschung einbringt (SL), die andere mit einem Studium der Wirtschaftsingenieurwissenschaft auf diesem Gebiet fachfremd ist, jedoch Sprach- und Kulturkenntnisse sowie Kontakte mitbringt, ohne die das Projekt nicht hätte verwirklicht werden können (SJ). Um einen direkten Einblick in die Situation von Kindern und Jugendlichen in den IDP-Camps in und um Kabul bekommen zu können, der über die mediale Berichterstattung hinaus geht, bot es sich an, onlinebasierte Expert_inneninterviews (vgl. Helfferich 2014) mit Helfer_innen vor Ort zu führen. Direkte Befragungen von Bewohner_innen der Camps wäre nicht nur schwerer umsetzbar gewesen, sondern auch aus forschungsethischer Sicht problematisch, da es sich um Menschen in akuter existentieller Not handelt. Als Feldzugang boten sich Kontakte an, die im Rahmen ehrenamtlichen Engagements zu einer kleinen lokalen Hilfsorganisation in Kabul bestanden. Die dort verorteten Menschen zeigten eine sehr hohe Motivation an der Mitwirkung an diesem Forschungsvorhaben, die sich nicht zuletzt im Interesse darin speiste, alle zur Verfügung stehenden Kanäle zu nutzen, um die internationale Aufmerksamkeit auf die Notsituation zu lenken.

## Mehrsprachige Interviewstudie

Wir verfassten eine Einverständniserklärung und einen teilstrukturierten Interviewleitfaden (vgl. Helfferich 2014) und übersetzten beide Dokumente in die Landessprache Dari. Auf Wunsch der Kooperationspartner_innen übersandten wir auch letzteren vorab an unsere Kontaktpersonen in Kabul. Daraus ergab sich die methodische Schwierigkeit, dass diese, die wir eigentlich als Interviewpartner_innen adressieren wollten, mit diesem Leitfaden ihrerseits ins Feld gingen und Kurzinterviews mit anderen Helfer_innen führten, die sie uns als Audiodateien zur Verfügung stellten. Diese transkribierten wir zunächst in der Originalsprache Dari und übersetzten sie anschließend ins Englische. Wir entschieden uns nicht für Deutsch, da Englisch eine Sprache ist, die uns gleichermaßen sicher zur Verfügung steht (und international anschlussfähig ist). Berg et al. (2019) weisen darauf hin, dass Mehrsprachigkeit in der Sozialforschung nie unabhängig von Machtverhältnissen – und auch nicht unabhängig von Kolonialismus – zu betrachten ist. Insofern entschieden wir uns für die Auswertung für eine Sprache, die für beide Autor_innen die Zweitsprache ist (Englisch), wobei die Erhebungssprache (Dari) nur von einem Autor gesprochen wird und die Publikationssprache (deutsch) von einer Autorin als Muttersprache und dem anderen Autor als fünfte Sprache erlernt wurde.

Da es sich bei der Interviewführung nicht um in der qualitativen Sozialforschung geschulte Personen handelte, wurden dabei wichtige Gesichtspunkte der Interviewführung, wie etwa das Anschmiegen an den Erzählfluss, das Aushalten von Pausen, das öffnende Wiederaufgreifen von kargen Antworten usw. (vgl. Helfferich 2014) nicht immer beachtet. Das Ergebnis waren daher zunächst recht kurze und „dünne“ Texte. Wir entschieden uns jedoch, auch diese Interviews in die Auswertung miteinzubeziehen. Damit sollte nicht nur die Varianz innerhalb des Samplings gestärkt werden. Darüber hinaus war uns auch wichtig, die Eigeninitiative im Sinne eines Partizipationsgedankens (auch wenn es sich dabei keineswegs um partizipative Forschung im eigentlichen Sinne handelt) zu würdigen. Dies war uns insbesondere wichtig, da die Konflikte aus Afghanistan nicht ohne die Perspektive postkolonialer Dynamiken zu verstehen sind, was eine besondere Sensibilisierung für den Umgang mit Deutungshoheiten und epistemischer Gewalt (vgl. Brunner 2020) erfordert. Um dennoch zu reicheren Interviewtexten zu kommen, führten wir im Nachgang selbst noch Interviews.

Das in den hier ausgeführten Ergebnissen berücksichtigte Sampling besteht aus drei jungen Frauen, die im Kontext lokaler Hilfsorganisationen humanitäre Unterstützung in IDP-Camps leisten. Zwei von ihnen haben einen Hochschulabschluss, eine befindet sich noch im Masterstudium. Frau Nouri^3^ und Frau Saidkhili wurden von unserer Kooperationspartnerin in Afghanistan interviewt. Das Gespräch mit Frau Hashemi, das mit Abstand das ausführlichste und längste war, führten wir (SJ) selbst.

## Auswertung

Die Arbeit mit qualitativen Interviews in mehr als einer Sprache und mehr als einem kulturell-politischen Kontext kann nach Reinke de Buitrago (2019) als „a challenging endeavor“ (o. S.) bezeichnet werden, die eine Reihe an Schwierigkeiten, wie die Gefahr von Missverständnissen, mit sich bringt. „Since researchers are all part of and thereby largely ‚caught‘ in their own language and cultural context, they may easily miss, misjudge or misinterpret meaning in another such context“ (ebd.).

Für die Auswertung unserer Interviews arbeiteten wir mit den aus Dari ins Englische übersetzten Transkripten. Diese waren, im Unterschied zu den orginalsprachlichen, für uns beide zugänglich. Hier ergibt sich durchaus eine Fehlerquelle, insbesondere, da es sich nicht um Übersetzungen durch eine_n professionell geschulten Dolmetscher_in handelte. Insofern konnten translationswissenschaftlich relevante Entscheidungen, wie etwa Übersetzungsstrategien nach dem um Ähnlichkeit bemühten Äquivalenzansatz oder dem pragmatisch orientierten Skopos-Ansatz nur intuitiv getroffen und nicht explizit gemacht werden (vgl. Brandmeier 2015; Enzenhofer und Resch 2013). Unter anderem aus diesem Grund gelten Interviews mit Übersetzungsleistung oft als zweitbeste Lösung, sind aber, wie in diesem Falle auch, oft der einzige Zugang zu Stimmen, die sonst ungehört blieben (Brandmaier 2015.

Als Auswertungsmethode wählten wir die zusammenfassend-induktive Variante der Qualitativen Inhaltsanalyse (vgl. Mayring und Fenzl 2014) aus. Obwohl wir nur eine kleine Datenmenge erhoben haben, erschien sie uns für den gegebenen Forschungskontext geeignet, da sie für den fachfremden Forschungspartner schnell erlernbar war. Zudem erschienen stärker hermeneutisch ausgerichtete Verfahren angesichts des besonders hohen Potentials für Unsicherheiten und Missverständnisse durch den Erhebungskontext weniger gut geeignet. Als Kodiereinheit definierten wir bedeutungstragende Phrasen, als Kontexteinheit den gesamten auf einen Interviewer_innenimpuls folgenden Textabschnitt und als Auswertungseinheit alle englischsprachigen Transkripte. Die Entwicklung und Benennung der Kategorien sowie die Formulierung der Kodierregeln erfolgte auf Englisch. Dabei konnten wir als Autor_innen unsere Heterogenität hinsichtlich sprachlich-kultureller Vorerfahrungen nutzen, um uns diskursiv über die Kategorien auszutauschen (vgl. Reinke de Buitrago 2019).

## Ergebnisse

Bei der Inhaltsanalyse wurden elf Kategorien gebildet. Aus Platzgründen werden im Folgenden die beiden Kategorien vorgestellt, in denen die meisten Textstellen aus allen drei Interviews, die im direkten Zusammenhang mit der Forschungsfrage stehen, subsummiert werden konnten: „Existentielle Not“ und „Übergriffe auf die sexuelle Selbstbestimmung“.

### Existentielle Not

Unter der Kategorie K2 „Existential need“ wurden Textstellen zusammengefasst, in denen von existentieller Armut, dem Fehlen von Nahrung, Kleidung, Wärme und Gesundheitsversorgung die Rede war. Als Ankerbeispiel diente uns die Textstelle:

the people who are living in these camps, unfortunately do not have anything from food to clothes and accommodation, and the basic facilities of life, they have nothing at all and this is the reason why they lost their lives and made it so terrible that they can not lead their lives well (Nouri Pos. 16-7).

Die Interviewten berichten davon, dass Menschen in den IDP-Camps aufgrund von Hunger, extremen Klimabedingungen (erst Hitze, im Winter droht Kälte) und fehlender Gesundheitsversorgung Gefahr von Leib und Leben ausgesetzt sind („Loss of life due to hunger, heat and poor health“, I3 Interview_Hashemi, Pos. 27). Die Menschen mussten bei der kriegsbedingten Flucht aus ihren Heimatdörfern sämtliches Eigentum zurücklassen. In den IDP-Camps haben sie weder die Möglichkeit, zu arbeiten, noch werden sie ausreichend versorgt. Sie leben in provisorischen Zelten.

Frau Hashemi erzählt vom Zelt einer siebenköpfigen Familie:

I deeply observed the whole tent with my own eyes. The roof of the tents was covered only in one part with a women’s scarf and a men’s handkerchief, which the rural men spread on their shoulders were used as a carpet on the hard and dusty ground. One or two pairs of the clothes they had brought with themselves were used as pillows (I3 (Interview_Hashemi): 14)

Besonders herausgestellt wird die fehlende Gesundheitsversorgung, die ebenfalls Menschenleben kostet: „there is no doctor to treat them, no medicine for them, no means of basic life to make them breathe a little with more comfort“ (I3 (Interview_Hashemi), Pos. 24). Als besonders gefährdet werden dabei Kinder und alte Menschen eingeschätzt. Insbesondere unter ihnen sind Todesfälle zu verzeichnen. Zwei der Interviewten berichten davon, dass sie mit eigenen Augen gesehen haben, wie Menschen an Unterernährung und fehlender medizinischen Hilfe verstorben sind.

Frau Hashemi erzählt zudem von einem Mann, dessen schwangere Frau im Camp zu früh niedergekommen sei und die Geburt nicht überlebt habe. Zur Trauer um seine Ehefrau kommt für ihn nun die Sorge, wie er sein neugeborenes Kind und dessen zwei Schwestern ernähren soll, da keine Ersatzmilch und kaum Nahrung zur Verfügung steht. Sie zitiert den verzweifelten Vater:

Hedyeh and Marjan, my two daughters, want their mother every moment. They were very used to their mother and I can not calm them down and I do not know how to take care of this newly born baby, because I have nothing for her. There is nothing we have; no milk, no clothes and no pumpers to prevent this newly born baby girl, whom I now call Mohajir (immigrant). I have sorrows to lose my daughters because of extreme poverty. I know that the soul of my wife Tamana is not calm with the situation that Marjan, Hadeyeh and Mohajir goes through, I ask you to be the reflector of our voices to be heard to all people to help us. (I3 (Interview_Hashemi): 20)

Neben zahlreichen weiteren Beispielen von Armut, Hunger und Not problematisieren die Interviewten, dass die Situation der Menschen in den IDP-Camps bei der neuen Regierung nur marginale Aufmerksamkeit erfährt und nicht ausreichend priorisiert wird.

On the other hand, the government is in such a predicament that it has no time at all to put the displaced in the spotlight and if the important tasks of the government are prioritized, I can say with regret and the utmost courage that their last priority will be to address the problems and shortcomings of the IDPs. The lack of attention of the authorities to the poor living conditions and lack of access to health services causes the elderly and children to resist with death and in the end, they can not resist and surrender to death (I3 (Interview_Hashemi), Pos. 27).

### Übergriffe auf die sexuelle Selbstbestimmung

In Kategorie 6 „threat to sexual self-determination“ sind Textstellen zusammengefasst, die darauf hinweisen, dass IDP-Camps insbesondere für Frauen und Mädchen hochgradig riskante Orte sind, an denen ihre sexuelle Selbstbestimmung gefährdet sind. Das Ankerbeispiel hier war:

and besides the mentioned facts, another *most terrible* thing is the forced marriages of the young girls against theit consent because the families want to secure their daughters at least in a way, therefore they are forcing (…) their daughters to get married. (I2 (Interview_Saidkhili): 27)

Das Spektrum der in dieser Kategorie genannten Gefährdungen beginnt allerdings schon beim Fehlen von einigermaßen sicheren Räumen zum Schutz der Intimsphäre, etwa für Toilettengänge, zum Waschen, für Hygienemaßnahmen während der Menstruation. Frau Hashemi weist darauf hin, dass diese Dinge insbesondere in der afghanischen Kultur hochsensibel sind und Mädchen und Frauen daher das – in den Camps nicht erfüllte – Bedürfnis nach Sicherheit und Schutz haben:

It is also very important to provide sanitary ware for women, which is not available to most of these women and girls. (…) On the other hand, the life in Afghanistan is such a way that women, especially a young girl, do not dare to talk to a man about their need to have personal belongings and ask for it, and now that they live in a camp, the environment is new to them and they do not know the place and do not have hygenic materials and it is very difficult (I3 (Interview_Hashemi): 39–40)

Auch Frau Saidkhili sieht in diesem Aspekt „very serious problem […]“. (I2 (Interview_Saidkhili): 37). Des Weiteren sind Mädchen und Frauen Voyeurismus ausgesetzt. Frau Hashemi schildert ihre Beobachtungen, wie Mädchen und Frauen, die aufgrund von Hitze ihre stickigen Zelte verlassen und sich an der frischen Luft aufhalten, Blicke von Männern auf sich ziehen, die sich ein Vergnügen daraus machen: „all their eyes were on the women and girls who were in these camps to pass thier time and to hunt these poor women and girls by their evil eyes“ (I3 (Interview_Hashemi): 22–23).

Alle Interviewten thematisieren aber auch, dass junge Mädchen zum Zwecke von Zwangsverheiratungen an Männer verkauft werden. So berichtet etwa Frau Nouri: „(…) and on other days when I went to the camp and wanted to survey the camp, I am witnessed and saw a father selling his two children so that he can earn some money and save other members of his family with this money“ (I1 (Interview_Nouri): 15). Frau Hashemi erzählt davon, wie ein Elternpaar von sich selbst sagt, sie hätten schweren Herzens die älteste Tochter geopfert. Sie zitiert die Mutter:

We made them [unsere Kinder, SJ&SL] go to school with a thousand hardships so that their future would be good. The sufferings we endured that at least our children live peacefully, unfortunately everything got worse, neither the house nor the land and property remained for us and the lives of our children became hell. The hunger of our children, their lack of clothes, their bare feet, the lack of blankets for their sleep, the illnesses of their father who has both sugar and kidney problems and as you see, he is like a dead body, there is no money for his medicine. All these tragedy has resulted that we have to give our 17-year-old daughter Zahra to a 45-year-old man for a small amount of money and accept her marriage to 45 years old Wahab in order to save her father, siblings and younger brothers from certain hunger deaths (I3 (Interview_Hashemi): 15–17)

Das betroffene Mädchen beschreibt Frau Hashemi als

an extremely oppressed and depressed girl, a girl that from her appearance can be sured that she still has her hopes for a life. Her colorless big eyes with full of tears, dry lips, bony and yellow face and thin body very clearly express her pain and sorrow. From her opresssed eyes, one can clearly observe that she hates life so much which seems to be doomed to breathe to her she has lost the way to take the burden of all this suffering to nowhere. (I3 (Interview_Hashemi): 15–17)

Das Mädchen erzählte Frau Hashemi unter Tränen davon, dass sie die elfte Klasse absolviert habe und sich auf ein Universitätsstudium vorbereiten wollte. Die Diabeteserkrankung ihres Vaters habe sie motiviert, ein Medizinstudium anzustreben. Doch nun seien diese Hoffnungen zunichte gemacht:

now my pain is not just the crooked back of my parents and the fear of my siblings loss due to hunger, not going to school and the destruction of my dream of becoming a doctor. Today, my pain is bigger and bigger than this: I have to marry a 45-year-old man and become the lustful toy of a man who has a wife and four children (I3 (Interview_Hashemi): 15–17).

## Fazit

Die dargestellten Interviewausschnitte geben erste Eindrücke davon, dass sich (junge) Menschen in Kabuler IDP-Camps mit Situationen konfrontiert sehen, die massive Risiken für ihre Entwicklung in jeder Hinsicht bedeuten und in manchen Fällen zur Gefahr für Leib und Leben werden können. Hunger sowie die fehlende Versorgung mit Nahrung, medizinischer Hilfe und weiteren Gütern des täglichen Lebens gefährden insbesondere die vulnerabelsten Gruppen wie kleine Kinder, Schwangere und alte oder kranke Menschen. Zusätzlich stellen sich für weiblich gelesene junge Menschen besondere Gefährdungsrisiken im Bereich der sexuellen Selbstbestimmung dar. Diese Ergebnisse sind besonders anschlussfähig an Studien zur Situation von Frauen in Flüchtlingslagern in anderen Ländern (vgl. Krause und Schmidt 2018). Weitere, hier nicht dargestellte Ergebnisse, weisen darauf hin, dass insbesondere der anstehende Winter und die psychologischen Folgen der Not Anlass zur Sorge geben. Die hier dargestellten Ergebnisse decken sich mit Fällen aus der Medienberichterstattung. Einschränkend muss zugestanden werden, dass das bisher vorliegende Datenmaterial noch von geringem Umfang ist und die Überbrückung von Sprachen und politisch-kulturellen Unterschieden forschungsmethodische Herausforderungen mit sich bringt. Dennoch bieten die Interviews die Möglichkeit, Stimmen, die der (deutschen) Sozialforschung nur schwer zugänglich sind, Gehör zu verschaffen und den Blick auch auf das subjektive Erleben innerhalb der kollektiven Not zu richten. Hier bedarf es weiterer Forschungsbemühungen, um die Sensibilität für (junge) Menschen in den Camps und ihre individuellen Schicksale aufrecht zu erhalten – und vor allem humanitäre Hilfe.

## Footnotes

1 Die Ereignisse wurden international in den Medien diskutiert und werden daher an dieser Stelle nicht näher ausgeführt.

2 Quelle: mündliche Informationen unserer Kontaktpersonen der lokalen Hilfsorganisation *Family Support and Welfare Organization* in Kabul.

3 Alle Namen sind pseudonymisiert. Aus Sicherheitsgründen tauchen hier auch keinerlei genauere Ortsangaben auf.
